# Time-of-flight photoelectron momentum microscopy with 80–500 MHz photon sources: electron-optical pulse picker or bandpass pre-filter

**DOI:** 10.1107/S1600577521010511

**Published:** 2021-11-03

**Authors:** G. Schönhense, K. Medjanik, O. Fedchenko, A. Zymaková, S. Chernov, D. Kutnyakhov, D. Vasilyev, S. Babenkov, H. J. Elmers, P. Baumgärtel, P. Goslawski, G. Öhrwall, T. Grunske, T. Kauerhof, K. von Volkmann, M. Kallmayer, M. Ellguth, A. Oelsner

**Affiliations:** a Institut für Physik, Johannes Gutenberg Universität, 55128 Mainz, Germany; b BESSY II, Helmholtz-Zentrum, 12489 Berlin, Germany; c MAX IV Laboratory, Lund University, PO Box 118, SE-221 00 Lund, Sweden; d APE GmbH, 13053 Berlin, Germany; e Surface Concept GmbH, 55128 Mainz, Germany

**Keywords:** time-of-flight spectroscopy, momentum microscopy, ARPES, photoelectron diffraction, pulse picking

## Abstract

The small time gaps of synchrotron radiation in conventional multi-bunch mode (100–500 MHz) or laser-based sources with high pulse rate (∼80 MHz) are prohibitive for time-of-flight (ToF) based photoelectron spectroscopy. Detectors with time resolution in the 100 ps range yield only 20–100 resolved time slices within the small time gap. Here we present two techniques of implementing efficient ToF recording at sources with high repetition rate. A fast electron-optical beam blanking unit with GHz bandwidth, integrated in a photoelectron momentum microscope, allows electron-optical ‘pulse-picking’ with any desired repetition period. Aberration-free momentum distributions have been recorded at reduced pulse periods of 5 MHz (at MAX II) and 1.25 MHz (at BESSY II). The approach is compared with two alternative solutions: a bandpass pre-filter (here a hemispherical analyzer) or a parasitic four-bunch island-orbit pulse train, coexisting with the multi-bunch pattern on the main orbit. Chopping in the time domain or bandpass pre-selection in the energy domain can both enable efficient ToF spectroscopy and photoelectron momentum microscopy at 100–500 MHz synchrotrons, highly repetitive lasers or cavity-enhanced high-harmonic sources. The high photon flux of a UV-laser (80 MHz, <1 meV bandwidth) facilitates momentum microscopy with an energy resolution of 4.2 meV and an analyzed region-of-interest (ROI) down to <800 nm. In this novel approach to ‘sub-µm-ARPES’ the ROI is defined by a small field aperture in an intermediate Gaussian image, regardless of the size of the photon spot.

## Abstract 2

The small time gap in normal multibunch operation of storage rings is prohibitive for high-resolution time-of-flight spectroscopy but this problem can be solved by two approaches. Electron-optical pulse picking by a GHz beam blanking unit can select any desired pulse period (phase locked to the storage ring) and bandwidth pre-selection reduces the energy band entering the time-of-flight analyzer.

## 1. Introduction

Thanks to its perfectly periodic time structure, synchrotron radiation is an ideal tool for time-of-flight (ToF) photoelectron spectroscopy (Becker & Shirley, 1996 ▸) real-space spectroscopic photoemission electron microscopy (ToF-PEEM) (Spiecker *et al.*, 1998 ▸; Oelsner *et al.*, 2001 ▸), angle-resolved ToF spectroscopy (Öhrwall *et al.*, 2011 ▸; Ovsyannikov *et al.*, 2013 ▸; Berntsen *et al.*, 2011 ▸) or momentum microscopy (MM) (Schönhense *et al.*, 2015*a* ▸). Covering a huge interval in (*E*_kin_,**k**) parameter space in a single exposure, the ToF-MM approach has proven powerful in the vacuum ultra-violet (VUV) (Chernov *et al.*, 2015 ▸), soft-X-ray (Medjanik *et al.*, 2017 ▸) and hard-X-ray range (Medjanik *et al.*, 2019 ▸; Elmers *et al.*, 2020*a* ▸). All these ToF-based methods establish a highly effective approach to angular-resolved photoelectron spectroscopy (ARPES), the method of choice for studies of the electronic structure (Hüfner, 2003 ▸; Reinert & Hüfner, 2005 ▸; Suga & Sekiyama, 2013 ▸). This branch of photoemission is steadily growing, being fueled by the discovery of topological states and exciting electronic properties of quantum materials (Lv *et al.*, 2019 ▸; Belopolski *et al.*, 2019 ▸; King *et al.*, 2021 ▸) and access to *in operando* devices (Hofmann, 2021 ▸).

Recently, the advent of full-field-imaging MM as a new approach to hard-X-ray photoelectron diffraction (hXPD) (Fedchenko *et al.*, 2019 ▸) has opened an avenue toward fast and effective recording of structural information. The high site specificity of hXPD (Fedchenko *et al.*, 2020 ▸) can be exploited as a unique fingerprint of atomic sites in compounds. The first applications of this young technique for the analysis of Mn dopant sites in the diluted ferromagnetic semiconductor (GaMnIn)As (Medjanik *et al.*, 2021 ▸) and Te in the Si lattice in the hyperdoped regime (Hoesch *et al.*, 2021 ▸) shine a first light on the potential of hXPD. Electronic and structural information can be obtained in a single experiment and under identical conditions (kinetic energy of the photoelectrons, size and position of the probing spot, probing depth). In turn, this allows eliminating the strong hXPD patterns imprinted on the valence-band momentum images in hard X-ray ARPES (HARPES), allowing mapping of intensity-corrected valence bands (Babenkov *et al.*, 2019 ▸; Schönhense *et al.*, 2020*a* ▸; Elmers *et al.*, 2020*a* ▸; Medjanik *et al.*, 2021 ▸). A time-resolved variant of ToF-MM with fs-pulsed sources was recently established at the free-electron laser FLASH at DESY, Hamburg, Germany (Kutnyakhov *et al.*, 2020 ▸; Dendzik *et al.*, 2020 ▸; Pressacco *et al.*, 2021 ▸).

The efficiency of the three-dimensional recording architectures of angular-resolved ToF spectrometers and MMs is defined by the time-resolving image detector. Delay-line detectors (DLDs) (Oelsner *et al.*, 2010 ▸; see also https://www.surface-concept.com/index.html; for multiline detectors see: https://www.surface-concept.com/downloads/info/ml_dld.pdf) or the upcoming solid-state pixel arrays (Zhao *et al.*, 2017 ▸; Giacomini *et al.*, 2019 ▸) are characterized by time resolutions in the 50–200 ps range. Hence, for ToF-based experiments a pulse period in the range 50–500 ns would be favorable, in order to provide a sufficiently long time gap for resolving ∼10^3^ time slices in a spectrum. The vast majority of experiments at synchrotron radiation sources requires maximum photon flux and do not need time structure. Hence, most synchrotron run times provide users with quasi-CW X-rays with pulse rates between 100 and 500 MHz, with limited time dedicated to ‘few-bunch’ modes for experiments that exploit the pulsed nature of the light. Corresponding periods of 2–10 ns are too short for high-resolution ToF-electron spectroscopy with a sufficient number of resolved times slices. The same arguments apply to the upcoming photon sources based on high harmonic generation (HHG) with intra-cavity conversion (Corder *et al.*, 2018*a* ▸,*b* ▸; Mills *et al.*, 2019 ▸) and for UV laser sources running at pulse rates in the 80 MHz range.

Up to now there are basically two approaches for the selection of longer periods between the photon pulses. The first approach is realized as an electron bunch separation scheme, where a single bunch or few bunches are displaced in the transversal plane from the main orbit and filling pattern by different techniques. At the ALS (Berkeley, USA), a high-repetition-rate vertical kicker magnet is used to displace one single bunch of the filling pattern to a different vertical orbit (Sun *et al.*, 2012 ▸). At BESSY II (Berlin, Germany), one single bunch of the filling pattern is blown up in the transverse dimensions by pulse picking resonant excitation (Holldack *et al.*, 2014 ▸). An aperture in the beamline blocks the radiation pulses from the main orbit, only selecting the blown up parts of the excited bunch, resulting in a reduced repetition rate, but with reduced intensity. Kicking the electrons with a selectable angle through an insertion device is also an important ingredient in femtosecond-slicing experiments (Schoenlein *et al.*, 2000 ▸). At BESSY II, another technique is under development, which is based on a special storage ring setting, generating a second stable ‘island orbit’, wiggling around the main orbit in the equatorial plane (Goslawski *et al.*, 2019 ▸). All these transverse bunch separation schemes provide two different radiation sources in bending magnets and undulators for the beamlines, which could be selected by apertures.

The second approach is a fast mechanical chopper for the radiation pulses within the beamline on the basis of a high-speed rotating disk with slits. The rotation frequency is synchronized with the storage-ring period which requires precise phase matching with the filling pattern of the storage ring. Pioneering work has been performed at the ESRF (Grenoble, France) (Cammarata *et al.*, 2009 ▸) and BESSY II (Förster *et al.*, 2015 ▸). In the latter experiment the so-called camshaft bunch (single bunch in a gap of the filling pattern) has been successfully used for angular-resolved ToF spectroscopy. This camshaft bunch is separated by a gap of 60 ns on both sides from the multi-bunch train, where the pulses have only 2 ns spacing. Mechanical chopping is highly demanding in terms of stability and synchronization. The chopper at BESSY II operates at a frequency of 1.25 MHz.

In this article we describe two novel methods that do not require an electron-bunch separation within the storage ring or the beamline. The first approach is based on *electron-optical pulse picking*, using a special ToF-MM. In a photoemission experiment the photon pulses are converted into a pulsed electron signal with the same time structure. For pulse picking, a fast electric beam deflector with gigahertz bandwidth is integrated between two pinholes in the imaging column of a photoelectron microscope. A time-resolving image detector, here a delay-line detector (DLD), is used for spatio-temporal beam diagnostics and for *k*-space and real-space imaging. Results are shown from two different experiments: photoelectron pulses generated by a soft-X-ray pulse train with 200 ns period (chopper frequency 5 MHz) have been selected out of the 100 MHz multi-bunch train at the (former) storage ring MAX II (Lund, Sweden) (Andersson *et al.*, 2002 ▸). Electron pulses excited by a VUV beam with 800 ns period have been used by picking the camshaft bunch of BESSY II. Full extinction of all undesired photoelectron bunches is reached by proper positioning of two beam crossovers in two pinholes in the electron path. When passing the fine pinholes, the momentum image is encoded in terms of the angular distribution of the electrons (reciprocal image). The performance of the experiment using the chopped camshaft pulse is compared with the conditions for a special 5 MHz island-orbit filling pattern of BESSY II.

An alternative way of chopping the electron pulse train in the time domain by a GHz deflector is pre-selection of a reduced bandpass in the energy domain. This confines the width of the electron spectrum entering the ToF analyzer, so that the full pulse rate can be retained. We studied this approach using a hemispherical analyzer as dispersive element in a setup with a ps-pulsed UV-laser (80 MHz pulse rate, <1 meV bandwidth). The two approaches have been characterized with different photon sources, synchrotron radiation with 100 and 500 MHz pulse rate (the latter in the camshaft hybrid mode) for the pulse-picking method *versus* 80 MHz pulsed laser excitation for the bandpass pre-filter. A first quantitative estimation indicates both approaches yielding similar performance.

The high photon flux of this laser allowed momentum microscopy with an energy resolution of 4.2 meV and a small analyzed region-of-interest (ROI). In this novel approach to ‘sub-µm-ARPES’ the ROI is defined by a small field aperture in an intermediate Gaussian image, regardless of the size of the photon spot. Measured ROI sizes of 1300 and 800 nm for 20 and 10 µm field apertures suggest potential of further improvement to ROIs < 500 nm. High-frequency pulse picking or bandpass pre-filtering can enable efficient ToF spectroscopy, momentum microscopy and real-space imaging with few-meV resolution and sub-µm spatial resolution at synchrotron radiation sources with standard multi-bunch filling patterns, highly repetitive lasers or cavity-enhanced HHG sources.

## 2. Experimental techniques and results

### 2.1. Layout of the fast electron-optical pulse picker

The measurements were carried out using soft X-ray pulses at beamline I 1011 (Kowalik *et al.*, 2010 ▸) of MAX II and VUV pulses at beamline U125-2_NIM (Baumgärtel & Packe, 2016 ▸) of BESSY II in normal multi-bunch operation. For comparison, a reference measurement was performed at BESSY II using a special ‘island orbit’ with a different filling pattern of four bunches, oscillating around the equatorial plane of the storage ring. This orbit is spatially separated from the common multi-bunch train; the synchrotron radiation from this few-bunch pulse train is selected by inserting an aperture at an intermediate focus of the beamline.

Beamline I 1011 at MAX II provided linearly, circularly and elliptically polarized X-rays in the range 0.2–1.7 keV. The source was an elliptically polarizing undulator of the APPLE II type with a period of 46.6 mm, installed on the 1.5 GeV MAX II storage ring, and the beamline was a collimated PGM design (Kowalik *et al.*, 2010 ▸). For the experiments presented here, linearly polarized photons with an energy of 500 eV were used, with an exit-slit size of 100 µm giving a FWHM of ∼200 meV at a photon energy of 500 eV.

Beamline U125-2_NIM at BESSY II comprises a 10 m normal-incidence monochromator providing linear polarized photons in the energy range 6–35 eV. Energy bandwidths of less than 10 meV can easily be achieved with the 300 lines mm^−1^ grating and entrance and exit slit sizes of 20 µm in the first order. Spectral impurities due to higher harmonics are effectively suppressed in the U125-2 undulator by its quasi-periodic structure of the magnets.

**Figure 1:**
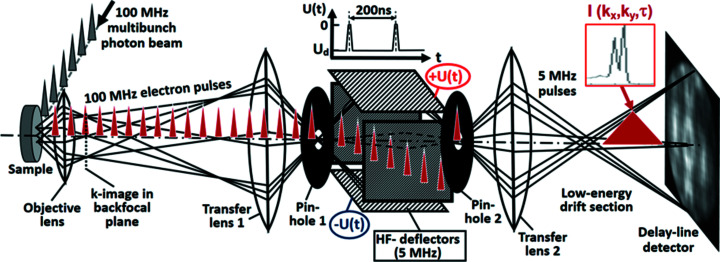
Schematic view of the electron-optical pulse picker in a time-of-flight momentum microscope operated at full multi-bunch filling of a storage ring. The depicted example corresponds to the situation at MAX II (100 MHz bunch pattern). The electron-optical system comprises three lens groups which transport and magnify the momentum image from the backfocal plane of the objective lens (left) to the delay-line detector (right). The sample emits a photoelectron pulse train of 100 MHz and each of the pulses (indicated by red triangles) contains the full spectrum. The high-frequency (HF) deflector is located between two narrow beam crossovers (pinholes 1 and 2). The deflector voltage *U*(*t*) (inset on top) deflects the electrons originating from the multi-bunch train, except for a short time interval where the voltage is zero, thus transmitting the electrons from the desired individual pulse through the second aperture (pinhole 2). This way, a 5 MHz sub-period of pulses is generated, which is dispersed in time-of-flight τ by the low-energy ToF section. The radial coordinate is strongly exaggerated; the screen shows a W 4*f*_7/2_ photoelectron diffraction pattern recorded at *h*ν = 500 eV. Note that each red triangle represents the full 3D array *I*(*k*_*x*_,*k*_*y*_,τ); in the pinholes the momentum pattern is encoded as angular distribution.

Fig. 1 ▸ shows the electron-optical scheme of the experiment. The high-frequency (HF) deflector is implemented into the column of a ToF MM. This special cathode-lens type electron microscope is optimized for best resolution in *k*-space and parallel acquisition of an energy interval of several eV width; for details, see Medjanik *et al.* (2017 ▸). MMs make use of the fact that a reciprocal image with high *k*-resolution and a linear *k*_∥_-scale occurs in the Fourier plane (backfocal plane) of the objective lens. The electron lenses in the microscope column project the magnified momentum image via a low-energy drift space for energy dispersion to the detector plane. The electron distribution in this plane is recorded by a 3D-resolving DLD (Oelsner *et al.*, 2010 ▸; see also https://www.surface-concept.com/index.html; for multiline detectors see: https://www.surface-concept.com/downloads/info/ml_dld.pdf). The pulse picker in the electron-optical column consists of *two pinholes* in two additional beam crossovers and the *HF deflector* placed in between. In the present experiment a simple quadrupole deflector was used; higher time resolution can be achieved with more sophisticated electrode arrangements. The deflector is supplied by coaxial lines with bandwidth 20 GHz (via SMB vacuum feedthroughs) and 50 Ω terminations. Fast voltage pulses with amplitudes up to 20 V are superimposed to the static potential of the deflector plates, centering the beam. The kinetic energy in the deflector is ∼100 eV. The HF generator is phase-locked to the clock of the pulsed photon beam, for example the bunch marker of the storage ring. Pinholes 1 and 2 are piezomotor-adjustable arrays of 16 size-selectable apertures, located in the planes of the beam crossovers.

**Figure 2:**
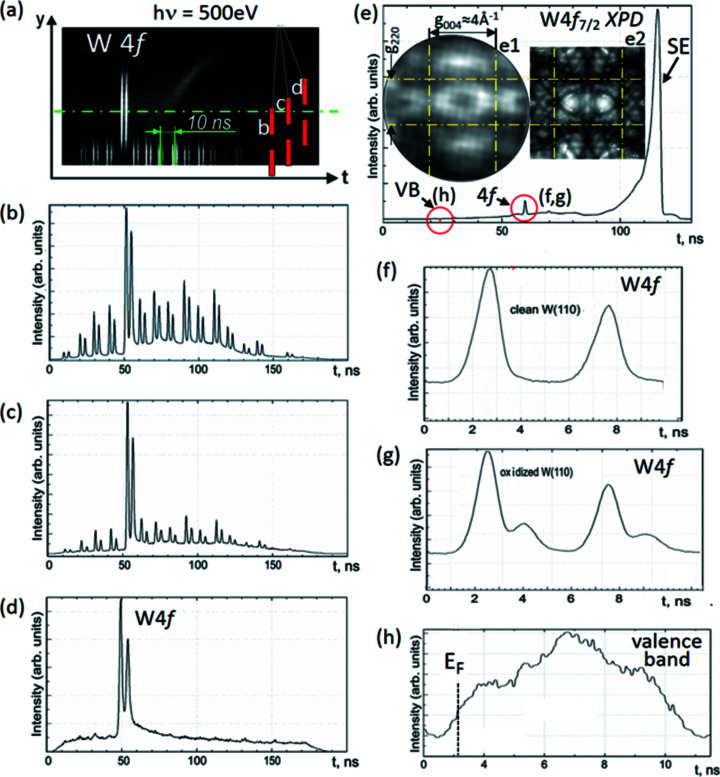
Operation of the HF deflector at 5 MHz at beamline I 1011 at MAX II (filling pattern 100 MHz); data recorded for a W(110) surface at *h*ν = 500 eV. (*a*) Spatio-temporal action visible in a measured *I*(*y*, *t*) cut through the 3D intensity distribution on the delay-line detector for fully opened *pinhole 2*. The two closely spaced lines correspond to the W 4*f*_7/2,5/2_ doublet, shifted along *y* by the fast deflector. (*b*, *c*, *d*) *I*(*t*) spectra with different degree of extinction, taken for three different positions of the selector aperture *pinhole 2* as indicated in (*a*). (*e*) Survey ToF spectrum (range ∼500 eV) recorded for complete extinction; the spectrum is strongly dominated by the secondary-electron peak (SE). Inset (*e*1): measured X-ray photoelectron diffraction (XPD) pattern for W 4*f*_7/2_ with the main Kikuchi bands being marked by dashed lines. Inset (*e*2): calculated W 4*f*_7/2_ XPD pattern. (*f*, *g*) W 4*f* core-level doublet and (*h*) valence-band spectrum taken at lower drift energy of 30 eV shown on a stretched time scale. The high background in the valence-band region (*h*) originates from higher-order contributions of the monochromator. Parts of the surface showed an oxidic overlayer (*g*).

The static and dynamic parts of the deflector voltages are adjusted such that the selected electron bunches with the desired period are transmitted through *pinhole 2*. All other electron bunches are blocked by deflecting them off-axis (*cf*. Fig. 1 ▸). The proper setting of the time interval is monitored by removing pinhole 2 and adjusting transfer lens 2 such that a real-space image of the plane of pinhole 2 is visible at the DLD at the end of the column [examples in Fig. 2 ▸(*a*) and 3 ▸(*b*)].

### 2.2. Pulse-picker operation with 5 MHz at MAX II (100 MHz filling pattern)

As an example, Fig. 2 ▸ shows a measurement of the tungsten 4*f* core-level and valence-band spectra, recorded at a photon energy of 500 eV at beamline I 1011 of MAX II. Fig. 2 ▸(*a*) shows an *I*(*y*, *t*) cut through the 3D spatio-temporal intensity distribution on the detector, recorded for fully opened *pinhole 2*. The pulse generator as well as the DLD (start signal) are phase-locked to the bunch marker of the storage ring. The two parallel lines in (*a*) represent the W 4*f* spin–orbit doublet, dispersed in the ToF section. This doublet shows up many times in the pattern, with spacing 10 ns, corresponding to a photon pulse rate of 100 MHz from the storage ring. It is eye-catching that one 4*f* doublet is located on the electron-optical axis (chain line), whereas all others are displaced off center by the static part of the deflector voltage *U*_d_. They are truncated by the rim of the image detector at the lower end of the panel. For this measurement, *transfer lens 2* was adjusted so that a real-space image is focused on the detector in order to visualize the 2D distribution.

*Pinhole 2* has been moved stepwise into the beam as sketched in Fig. 2 ▸(*a*), positions (*b*, *c* and *d*). In the Fig. 2 ▸(*b*) spectrum, taken with off-center pinhole, we observe the full multi-bunch train of MAX II with a period of 10 ns. The drop toward the rims of the time window is an artifact due to the trigger mode of the DLD. Moving the selector aperture to the next position yields a spectrum with partial extinction, Fig. 2 ▸(*c*), and position in Fig. 2 ▸(*d*) yields almost total extinction, with a small residual intensity from the other bunches. Changing transfer lens 2 to the momentum-imaging mode and setting a high drift energy leads to a wide-range spectrum, Fig. 2 ▸(*e*), with full extinction of undesired signals.

The temporal dispersion in the electron-optical column upstream of *pinhole 1* is very small and hence negligible. The HF deflector operates achromatic in a large energy range of several 100 eV width. Fig. 2 ▸(*e*) shows the complete photoemission spectrum from the low-energy cutoff (*E*_kin_ = 0) at 118 ns via the W 4*f* signal at 60 ns (*E*_kin_ = 460 eV) to the Fermi-energy cutoff at 22 ns (*E*_kin_ = 495 eV), recorded at fixed settings in a single exposure. The temporal dispersion dτ of the ToF drift section of length *L*_d_ depends strongly on the drift energy *E*_d_, according to

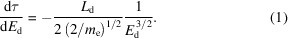

Due to the large variation of the drift energy between the low-energy cutoff at *E*_d_ = 30 eV and the Fermi cut-off at *E*_d_ = 525 eV, the temporal resolution varies from 31 ps eV^−1^ to 2.2 ns eV^−1^, respectively. Given the time resolution of the DLD (150 ps), the 4*f* doublet is not resolved in the survey spectrum, Fig. 2 ▸(*e*), but is well resolved when recorded at lower drift energy, Figs. 2 ▸(*f*) and 2(*g*).

In the *k*-imaging mode the chromatic aberration of the lens system limits the depth of focus to an energy band of up to 10 eV, depending on the size of the field aperture, which acts as a contrast aperture for *k*-imaging. It is a special property of the momentum microscope that different energy regions in a spectrum can be selected to be in focus by tuning just one parameter, namely the sample bias. The width of the spectrum can be restricted to a region of less than 10 eV below *E*_F_ by operating a section of the optics as a high-pass filter (for details, see Tusche *et al.*, 2016 ▸). However, this comes at the expense of the depth of focus because in the region of the saddle point the lens aberrations are very large.

The valence-band and core-level spectra in Figs. 2 ▸(*f*)–2(*h*) have been recorded on an expanded time-of-flight scale by selecting the proper drift energies in the ToF section. The valence-band spectrum is rather noisy since it suffers from a large background originating from higher-order contributions in the photon beam. The much stronger W 4*f* signal is very pronounced. Part of the surface is covered by chemisorbed oxygen, as visible in the well known chemical shift of the W 4*f* peaks in Fig. 2 ▸(*g*).

The W 4*f* core-level signal can be used for full-field imaging of the X-ray photoelectron diffraction (XPD) pattern (Fedchenko *et al.*, 2019 ▸). When the electrons are crossing the small selector aperture (*pinhole 2*), the XPD pattern is encoded in terms of their angular distribution. As an example the W 4*f*_7/2_ XPD pattern of the W(110) surface is shown as inset (e1) in Fig. 2 ▸. The characteristic diffraction pattern is clearly visible without significant distortion in the 5 MHz pulse-picking mode. The finite size of the selector aperture does not influence the resolution in the XPD image because for the angle-encoded diffraction pattern a small parallel shift of the beam inside of the aperture (∼100 µm) is insignificant for the momentum image on the DLD (40 mm diameter). A calculated W 4*f* XPD pattern for W(110) is shown in inset (e2); for details on the Bloch-wave model, see Fedchenko *et al.* (2019 ▸, 2020 ▸) and Medjanik *et al.* (2021 ▸). The calculated pattern shows many more details, which reflects that at 500 eV the Bloch-wave model is at its lower limit of the validity range. Similarities of measured and calculated patterns in the central horse-shoe-shaped patterns and dark regions allow assigning the principal Kikuchi bands corresponding to reciprocal lattice vectors *g*_004_ (vertical band) and *g*_220_ (horizontal band), as marked in Fig. 2 ▸(*e*).

### 2.3. Picking the ‘camshaft’ pulse with 1.25 MHz at BESSY II (at 500 MHz filling)

The pulse-picking approach can be used not only for ToF spectroscopy, real-space imaging and photoelectron diffraction but also for *momentum imaging of valence bands*. This application was explored at the storage ring BESSY II using the ‘hybrid’ filling pattern, consisting of a 500 MHz multi-bunch train plus a solitaire pulse (usually with higher bunch charge) in a gap of ∼120 ns width. The separation of this ‘camshaft’ pulse from the multi-bunch train is less demanding than the complete extinction of the neighboring bunches in the 100 MHz pulse train of MAX II. The relatively large time gap on both sides of the solitaire pulse relaxes the requirement for the slope of the leading and trailing edges of the blanking signal. Electrostatic gating of the camshaft pulse at BESSY II has previously been shown using a blanker electrode in a PEEM (Nickel *et al.*, 2013 ▸) and gating of the MCP-detector was applied in an angle-resolved ToF spectrometer (Stråhl­man *et al.*, 2016 ▸). In the present experiment we used a rectangular blanking signal *U*(*t*) of ∼700 ns length.

**Figure 3:**
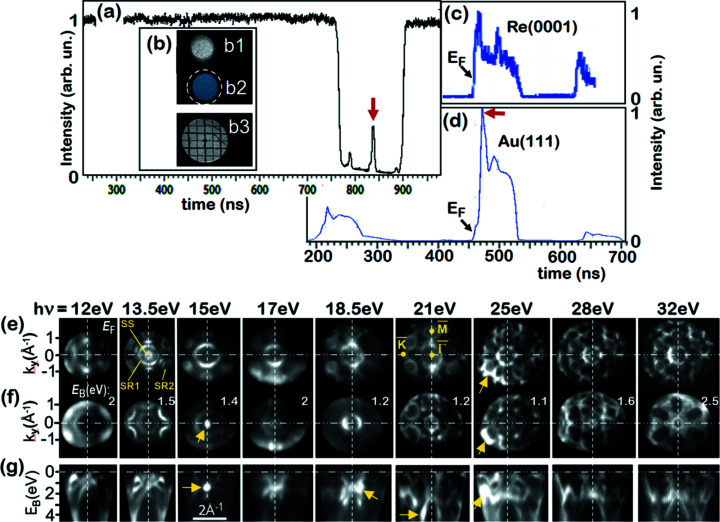
Time-of-flight spectra and momentum patterns recorded at BESSY II (U125-2_NIM) in multi-bunch hybrid-mode (with camshaft pulse). The spectrum of Au(111) without HF deflector (*a*) is dominated by the multi-bunch pulse train; the camshaft pulse (arrow) is visible in the center of the gap at 840 ns. After activating the HF deflector as blanking unit (*b*), the multi-bunch signal (*b*1) and camshaft signal (*b*2) are separated and the former is suppressed by the selector aperture, dashed circle in (*b*2). The auxiliary grid (*b*3) is located in the backfocal plane of the objective and serves for precise adjustment of the *k*-image on the delay-line detector. The time spectra (*c*, *d*) for Re(0001) and Au(111) are reduced to the camshaft pulse plus two adjacent small solitaire pulses at the lower and upper edge of the time gap, serving for reference (different filling for Re and Au). On the expanded time scale of (*c*, *d*) the ‘pulses’ appear as full valence-band spectra. Rows (*e*–*g*) show momentum sections through data arrays *I*(*E*_B_,*k*_*x*_,*k*_*y*_), recorded for Re(0001) in the pulse-picking mode at photon energies between *h*ν = 12 and 32 eV as marked on top. (*e*–*g*) *k*_*x*_–*k*_*y*_ cuts at *E*_F_ (*e*) and other binding energies as given in panels (*f*) and corresponding *E*_B_-*versus*-*k*_*x*_ sections (*g*) showing the band dispersions. In (*e*), SS, SR1 and SR2 mark a surface state and two surface resonances. 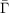 , 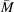 and 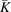 denote high-symmetry points in the surface Brillouin zone; arrows mark regions of local intensity enhancement by photoelectron diffraction.

Fig. 3 ▸ shows measurements for the Au(111) and Re(0001) surfaces recorded in the low-energy range (*h*ν = 12–32 eV) at beamline U125-2_NIM of BESSY II. The chosen exit slit of 100 µm yields a photon bandwidth of only 14 meV at *h*ν = 21 eV. The full time spectrum (referenced to the bunch marker signal) without blanking is shown in Fig. 3 ▸(*a*). The gap in the multi-bunch train spans from 760 to 900 ns. The camshaft pulse (arrow) appears at 840 ns and is framed by two additional smaller pulses at the rims of the time gap. After switching on the HF deflector, the signals from the multi-bunch train and camshaft pulse are separated in the plane of the selector aperture as visible in the real-space images of the field aperture (selector aperture fully open) in Fig. 3 ▸(*b*). The image originating from the multi-bunch train (b1) is shifted upwards [deflector voltage *U*(*t*) = *U*_d_ in Fig. 1 ▸], whereas the image generated by the camshaft pulse (b2) remains unshifted [deflector voltage *U*(*t*) = 0]. After driving in the selector aperture by the piezo motors (at the position of the dashed circle), the multi-bunch train is completely extinguished. Images b1 and b2 have been recorded by adjusting transfer lens 2 to real-space imaging. After checking the proper position of the selector aperture, transfer lens 2 is switched to momentum imaging. For rapid optimization an auxiliary grid is driven into the *backfocal plane* of the objective lens [dashed line left-hand side of Fig. 1 ▸]; the image of this ‘*k*-grid’ is shown in b3.

The time spectra in Fig. 3 ▸(*c*) for Re(0001) and Fig. 3 ▸(*d*) for Au(111) show just the camshaft pulse plus the two additional smaller pulses, purposely left in the recorded time window for calibration. On the stretched time scale the electron ‘pulses’ reveal their true nature. They represent complete spectra, here of the Re and Au valence bands. The repetition rate of the camshaft pulse is 1.25 MHz (period 800 ns); hence the time scale can be strongly stretched such that many time slices are resolved. The Re measurements were made in standard operation of BESSY II, where the camshaft-pulse current is ∼4 mA. This yields maximum count rates of >10^6^ counts s^−1^, being a typical condition for ToF MM in the VUV range. The single-channel DLD can record several 10^6^ counts s^−1^, a novel multi-line DLD (see https://www.surface-concept.com/index.html; for multiline detectors see https://www.surface-concept.com/downloads/info/ml_dld.pdf) will allow for 10^7^–10^8^ counts s^−1^. We notice that DLDs have a fast single-event counting scheme, which is crucial for measurements in the ultrafast time domain (Kutnyakhov *et al.*, 2020 ▸; Dendzik *et al.*, 2020 ▸; Curcio *et al.*, 2021 ▸; Pressacco *et al.*, 2021 ▸). The Au measurements were made in low-α multi-bunch mode with a reduced total beam current of 80 mA and a fraction of only 1 mA in the camshaft bunch. In this mode with shorter photon pulses (10 ps), the corresponding count rate with HF deflector was >10^5^ counts s^−1^ (photon bandwidth 14 meV).

The sequence of momentum patterns in Figs. 3 ▸(*e*)–3(*g*) show various sections through the measured 3D *I*(*E*_B_, *k*_*x*_, *k*_*y*_) data arrays recorded in the valence band of Re at photon energies between 12 and 32 eV. The full sequence was taken with increments of 1 eV (partly 0.5 eV) at typical acquisition times of 20 min for each photon energy. Rows (*e*) and (*f*) show *k*_*x*_–*k*_*y*_ sections at *E*_F_ and at higher *E*_B_ as given in the panels. Row (*g*) shows the corresponding *E*_B_-*versus*-*k*_*x*_ sections, revealing the band dispersions.

It is eye-catching that the patterns vary significantly with photon energy. In a MM the *k*-scale stays constant when varying the kinetic energy. Thanks to the high extractor field, the objective lens is achromatic in a wide range. The microscope records *all* emitted photoelectrons between *E*_kin_ = 0 and *E*_F_ in the full half space (2π solid angle). The size of the observed *k*-region increases with photon energy. Its outer rim in (*e*, *f*) is given by the photoemission horizon (emission angle θ = 90°). In momentum space the horizon corresponds to a certain radius of the observed *k*_||_-region in *k*-space. The radius at *E*_F_ (*E*_B_ = 0) increases from 1.35 to 2.7 Å^−1^ if *h*ν is varied between 12 and 32 eV.

Due to photoelectron refraction at the surface barrier, the horizon observed on the vacuum side corresponds to an (inner) escape cone of less than 90°. The *k*_||_-momentum is conserved when the photoelectron crosses the surface barrier. The momentum vector *k*_⊥_ of photoelectrons inside the material emitted into the direction of the surface normal varies from 2.40 to 3.32 Å^−1^ for *h*ν = 12 to 32 eV. In turn, the observed band pattern changes with photon energy, because the photo-transition leads to different final states of the periodic band scheme in 3D *k*-space. Finally, the patterns exhibit some regions of enhanced intensity due to photoelectron diffraction, intrinsic in the photoemission process, as discussed by Schönhense *et al.* (2020*a* ▸).

The high-symmetry points 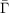 , 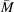 and 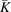 of the hexagonal-closed-packed surface Brillouin zone (BZ) as well as a Tamm surface state (SS) and two surface resonances (SR1 and SR2) are denoted in (*e*). The intensity from the surface state rapidly decreases with increasing residual gas exposure. Because some of the data were not acquired immediately after the flash cleaning, this explains its faint appearance in some of the measurements. In this energy range both surface and bulk bands are observed. The spin-polarized band structure of Re has been studied by Elmers *et al.* (2020*b* ▸), where all details of the band patterns are discussed. Here we just show the suitability of the pulse-picking mode for mapping of the (3D and 2D) BZ. Tomographic scanning of the bulk BZ requires variation of the photoelectron energy *E*_final_ (inside the material), which determines the radius of the final-state energy isosphere [for details, see Medjanik *et al.* (2017 ▸)]. Given the reciprocal lattice vector *G*_0001_ = 1.40 Å^−1^ for Re and assuming an effective mass of *m* = *m*_e_ and an inner potential of ∼11.5 eV, the present photon energy range corresponds to sections through the bulk BZ between 1.7 and 2.4*G*_0001_. Since the center (Γ-point) and the border of the BZ (A-point) lie at 2.0 and 2.5*G*_0001_, respectively, the range of *h*ν = 12–32 eV covers a large range of the bulk BZ. The Γ-point (*k*_*z*_ = 2*G*_0001_) was identified at *h*ν = 18.5 eV. For Au(111) the reciprocal lattice vector perpendicular to the surface is *G*_111_ = 2.68 Å^−1^. Here the photo-transition at *h*ν = 17 eV (corresponding to *k*_final_ = 2.66 Å^−1^) leads to the Γ-point of the first repeated BZ (*k*_*z*_ ≃ *G*_111_).

### 2.4. Bandpass pre-selection in the energy domain *versus* chopping in the time domain

In the previous sections we have introduced a method for pulse-picking of the electron signal in order to increase the time gap between adjacent electron pulses. Fast beam blanking reduces the fraction of analyzed electron pulses by one to two orders of magnitude. However, 3D parallel recording of *I*(*k*_*x*_, *k*_*y*_, *E*_B_) data arrays via ToF MM finally gains two to three orders of magnitude in recording efficiency. In turn, the net total gain is typically an order of magnitude.

Instead of increasing the time gap at the expense of the amount of analyzed pulses, we can reduce the analyzed energy band, retaining the full number of pulses in the multi-bunch train. This alternative approach requires the integration of an imaging dispersive energy filter for bandpass pre-selection. Both methods will be used in upcoming setups implementing efficient ToF parallel energy recording at synchrotron-radiation sources (Diamond, PETRA-III) in full multi-bunch mode.

For the selection of a well defined electron-energy bandwidth we use a single hemispherical analyzer (HSA) operated as momentum microscope. For optimum recording efficiency, the energy interval transmitted by the HSA is selected such that the desired resolution is reached for a maximum number of time slices resolved by the ToF analyzer behind the exit slit. The width of a single time slice after ToF dispersion defines the energy resolution. The new generation of DLDs provides a time resolution of ≤100 ps. This yields the following numbers of resolved time slices: *N* = 20, 50 or 100 for synchrotron sources with 500, 200 or 100 MHz (corresponding time gaps 2, 5 or 10 ns), respectively.

The feasibility of the *dispersive-plus-ToF hybrid mode* on the basis of a large HSA (450 mm diameter of the central beam) with ToF section behind the exit slit was proven by Schönhense *et al.* (2020*b* ▸). Here we present quantitative results and give a comparison of the recording efficiency of the HSA with and without the ‘ToF booster’. Finally, we compare the expected efficiencies of pulse-picking in the time domain by a fast HF deflector and bandpass pre-selection in the energy domain by a dispersive filter.

**Figure 4:**
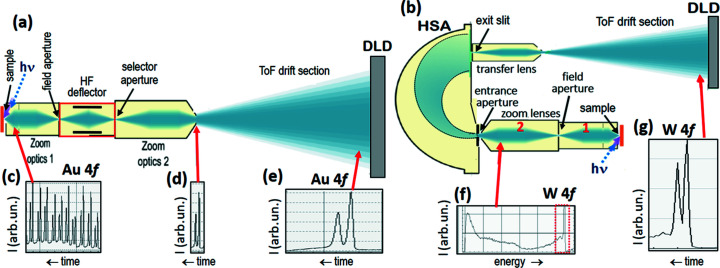
Two ways of implementing time-of-flight recording at highly repetitive photon sources. The first is based on pulse-picking in the time domain by an HF deflector (*a*), the second on bandpass pre-selection in the energy domain by a dispersive element (*b*). Electron-optical elements and ray bundles schematic. In (*a*) the multi-bunch train of almost-overlapping spectra [Au 4*f* doublet (*c*)] is blanked by the HF deflector so that a single spectrum (*d*) with larger period passes the selector aperture. Spectrum (*e*) with the desired resolution is gained by ToF dispersion in the low-energy drift section. In the dispersive-plus-ToF hybrid instrument (*b*), the hemispherical analyzer (HSA) cuts a well defined bandpass [here W 4*f* doublet] from the full energy spectrum (*f*). The transmitted energy band is precisely selected via pass energy and slit widths. This pre-selected energy band is dispersed in the ToF drift section, leading to spectrum (*g*). In both cases the various lens groups can project either a *momentum pattern* or a *real-space image* on the delay-line detector (DLD). Auxiliary grids (retractable) in the backfocal plane of the objective lens and in the plane of the field aperture enable precise adjustment of the lens optics.

Fig. 4 ▸ shows schematic views of the setups for pulse-picking (*a*) and dispersive pre-selection by an HSA (*b*). The multi-bunch photoelectron signal, here the Au 4*f* core-level doublet (*c*), is blanked by the HF deflector so that only a single spectrum remains (*d*), which is repeated with a larger period. This single spectrum is dispersed in the low-energy drift section, leading to the final spectrum (*e*). In the dispersive + ToF hybrid (*b*) the energy spectrum (*f*), here recorded for tungsten, is dispersed in the HSA (operated as MM). The exit slit transmits only a small, well defined energy interval. This interval is finally dispersed in time by the low-energy ToF drift section, leading to spectrum (*g*). The desired bandwidth transmitted by the exit slit can be precisely set via the pass energy and HSA slit widths. The subsequent ToF analysis cuts the energy band into *N* slices (*N* typically between 20 and 100). Notice that a pure ToF analysis, without the pre-filter, is not possible because full spectra are far too broad to be resolved with the low number of time slices.

One might ask whether ToF recording bears an advantage, when anyway a dispersive analyzer is used. The answer requires a deeper look into details of dispersive and time-of-flight energy discrimination and will be elucidated in the next section. In fact there is a substantial gain in recording efficiency when using the ‘ToF booster’ behind the exit slit. The reason lies in the increase of the dimensionality of the data recording scheme from 2D (in the conventional HSA mode as well as in HSA-based MMs) to 3D, when implementing parallel energy recording via ToF detection.

### 2.5. Proof-of-principle of bandpass pre-selection using a UV laser with 80 MHz pulse rate

The basic scheme and details of the dispersive-plus-ToF hybrid momentum microscope on the basis of a single-hemisphere HSA are described by Schönhense *et al.* (2020*b* ▸). Here we discuss and quantify the gain in recording efficiency by experiments using a high-repetition-rate UV laser [5.8–6.5 eV; bandwidth <0.5 meV; 10 ps pulse width; 80 MHz pulse rate (APE’s Levante Emerald ps OPO and HarmoniXX FHG (3+1) harmonic generator; https://www.ape-berlin.de/en/laser-arpes/)]. The extractor field collects all photoelectrons in the full solid-angle range of 2π, leading to a paraboloid-shaped *I*(*k*_*x*_, *k*_*y*_, *E*_kin_) 3D data array. The present single-channel DLD (180 ps time resolution) resolves 70 time slices in the 12.5 ns gap between adjacent laser pulses. For the evaluation we used *N* ≃ 50 slices neglecting the time slices close to the rims of the time gap in order to avoid temporal overlap of adjacent pulses.

**Figure 5:**
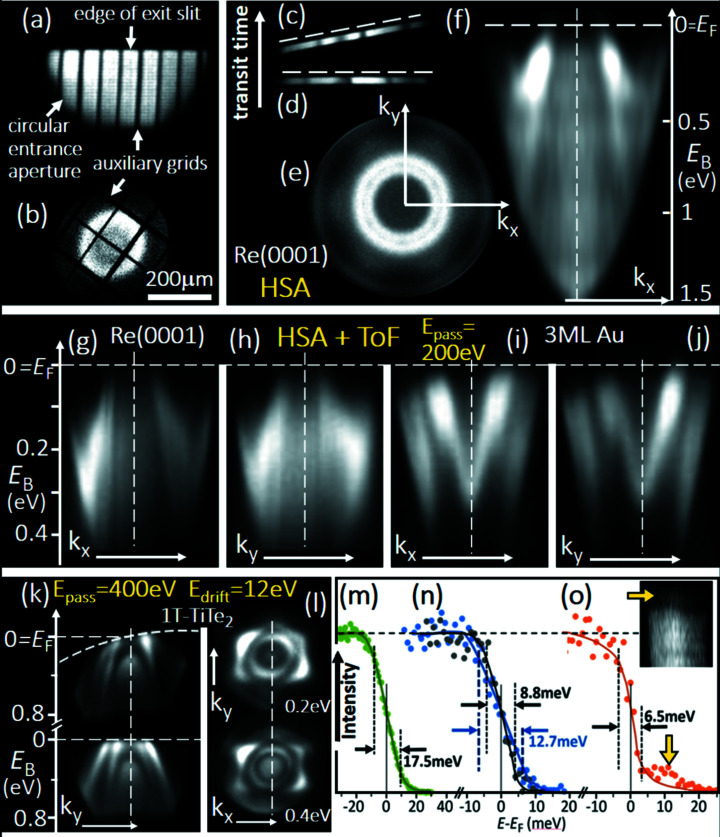
Measurements using the bandpass pre-selection approach, recorded with the setup shown in Fig. 4 ▸(*b*) using a narrow-bandwidth UV laser [*h*ν = 6.4 eV; bandwidth < 0.5 meV; 80 MHz (https://www.ape-berlin.de/en/laser-arpes/)]. (*a*) Gaussian image showing the superposition of the upper edge of the entrance slit, the circular entrance aperture (half circle) and the grid in the field-aperture plane (vertical lines). (*b*) Momentum image showing the superposition of the grid in the backfocal plane of the objective lens with a surface state. (*c*, *d*) Linear increase of transit time *versus* entrance angle in the dispersive plane (*c*) and constant behavior in the perpendicular (non-dispersive) plane (*d*), measured in the HSA + ToF hybrid mode at a drift energy of 500 eV. (*e*, *f*) Data array recorded using the classical scanning mode of the HSA without ToF analysis: *k*_*x*_–*k*_*y*_ momentum pattern of the Re(0001) Tamm state close to *E*_F_ (*e*) and *E*_B_-*versus*-*k*_*x*_ section of the full photoemission paraboloid with diameter ∼1.25 Å^−1^ at *E*_F_ (*f*). Second row, data recorded in the hybrid mode with *E*_pass_ = 200 eV and *E*_drift_ = 9 eV: (*g*, *h*) *E*_B_-*versus*-*k*_*x*,*y*_ cuts through data arrays of Re(0001) and (*i*, *j*) same for 3 ML of Au on Re(0001). (*k*, *l*) Data array (width 880 meV) for 1T-TiTe_2_, recorded in the hybrid mode at *E*_pass_ = 400 eV. (*k*) *E*_B_-*versus*-*k*_*y*_ section as-measured (top) and after numerical elimination of the transit time spread (bottom). (*l*) *k*_*x*_–*k*_*y*_ sections at *E*_B_ = 0.2 (top) and 0.4 eV (bottom). (*m*–*o*) Resolution measurements in the conventional scanning mode: intensity profiles of the Fermi edge of an Au film recorded at *E*_pass_ = 12 eV / *W* = 0.5 mm / *T* = 30 K (*m*), 8 eV / 0.4 mm / 30 K (blue) and 15 K (black) (*n*), and 8 eV / 0.2 mm / 15 K (*o*). Curves in (*m*,*n*,*o*) are to guide the eye; yellow arrows in (*o*) and the inset mark an intensity artifact slightly above *E*_F_.

The results of this pilot study are summarized in Fig. 5 ▸. Sample work functions were ∼5 eV, yielding arrays with depth 1.5 eV and diameter ∼1.25 Å^−1^ at *E*_F_. Figs. 5 ▸(*a*)–5(*h*) show data for the Tamm surface state of Re(0001), (*i*, *j*, *m*–*o*) data for the Shockley state plus quantum-well states on a three-monolayer film of Au(111) on Re(0001), and for the valence band of 1T-TiTe_2_ (*k*, *l*). Figs. 5 ▸(*e*, *f*, *m*–*o*) show measurements without ToF recording; all other results are recorded using the HSA+ToF hybrid mode.

*Without ToF recording*, the slit width *W* and pass energy *E*_pass_ of the HSA have to be set sufficiently small in order to reach the desired energy resolution. Neglecting the α^2^ term, the theoretical resolution of an HSA with central beam radius *R*_0_ is given by Δ*E*_theo_ ≃ *E*_pass_ *W*/2*R*_0_. Typical values for the present instrument are *W* = 0.5 mm and *E*_pass_ between 25 and 8 eV (yielding Δ*E*_theo_ between 28 and 9 meV). For high-resolution measurements, *W* can be reduced to 0.2 mm, yielding Δ*E*_theo_ = 11–2 meV for *E*_pass_ = 25–4 eV.

In the single-hemisphere MM two crucial adjustment conditions must be fulfilled (which is easier than the situation for a double-hemisphere instrument). First, there must be Gaussian (real-space) images in the entrance- and exit-slit planes of the HSA. This is facilitated by shifting an auxiliary grid into the first Gaussian plane [‘field aperture’ in Fig. 4 ▸(*b*)] and focus its image to the entrance plane of the HSA. Proper setting of the Jost electrodes ensure that this image is transferred 1:1 to the exit plane, an important precondition for good energy and momentum resolution. Fig. 5 ▸(*a*) shows a snapshot of the Gaussian image for a well adjusted optics: the edge of the exit slit (upper border), the rim of the circular entrance aperture (semi-circle, recorded at *E*_F_) and the lines of the auxiliary grid in the field-aperture plane are all in focus. Here the transfer lens behind the HSA exit is adjusted to form a Gaussian image on the detector. Second, the momentum image is optimized shifting a second auxiliary grid into the first reciprocal image plane (backfocal plane of the objective lens). Fig. 5 ▸(*b*) shows a snapshot of the *k*-image for a well adjusted optics, where the sharp shadow image of the *k*-grid is superimposed on the momentum image of a surface state (bright ring). Both conditions need to be fulfilled simultaneously, which requires successive optimization in several steps.

In order to avoid downgrading of the resolution by the α^2^ term in these first experiments, we limited the entrance angle into the HSA to α_max_ = 2° by appropriate setting of zoom optics [‘2’ in Fig. 4 ▸(*b*)]. An example recorded with *E*_pass_ = 25 eV / *W* = 0.2 mm (Δ*E*_theo_ = 11 meV) is shown in Figs. 5 ▸(*e*) and 5(*f*). In this mode without the ToF booster, the data array of 1.5 eV width has been acquired by taking (*k*_*x*_, *k*_*y*_) patterns sequentially, varying *E*_kin_ in steps of 5 meV (300 exposures of 20 s, total 1.7 h). The individual (*k*_*x*_, *k*_*y*_) patterns are finally concatenated yielding the 3D *I*(*k*_*x*_, *k*_*y*_, *E*_kin_) data stack.

*When activating the ToF booster*, the pass energy can be increased while retaining the energy resolution. Fig. 5 ▸ shows results for *E*_pass_ = 200 eV (*g*–*j*) and 400 eV (*k*, *l*), both with slits *W* = 1 mm, running the ToF drift section at *E*_drift_ = 9 eV. The as-measured *I*(*k*_*x*_, *k*_*y*_, τ) data arrays contain the contribution of the transit-time spread of electrons running on different Kepler ellipses in the HSA. The linear time-spread function is directly observable by running the ToF section at high drift energy, where the time dispersion is negligible (here *E*_drift_ = 500 eV). We observe a linear increase of the transit time with increasing momentum component *k*_*y*_, *i.e.* with increasing entrance angle α in the dispersive plane, Fig. 5 ▸(*c*). As expected, there is no time spread as a function of the entrance angle in the non-dispersive plane, Fig. 5 ▸(*d*); in both cases the entrance angle varies between α = −2° and +2°. At *E*_drift_ = 9 eV the ToF analyzer disperses the transmitted energy band of 880 meV width for *E*_pass_ = 400 eV in time by 8.86 ns. Assuming a time resolution of 180 ps, this results in ∼50 resolvable slices of 18 meV spacing.

The combination of the high pass energy of the HSA and the subsequent parallel acquisition of many energy slices via ToF analysis leads to a substantial increase of the recorded intensity. The efficiency gain is defined by the reduction of acquisition time needed to record the same data set with identical statistics, *i.e.* total number of counts in the *I*(*k*_*x*_, *k*_*y*_, *E*_kin_) array and identical energy resolution. The key factor is the transmission of the HSA, which is defined by the size of the entrance slit (restricting the fraction of the electron beam which enters the analyzer) and exit slit (restricting the transmitted energy band). When increasing the pass energy by a factor of *N*, the bandwidth increases by this factor. Comparing the scanning mode and the HSA + ToF hybrid mode at identical resolution, the gain due to the wider energy band passing the exit slit equals the number of resolved time slices *N*. The contribution of the entrance aperture is more complex and depends on several factors. The entrance plane hosts a magnified image of the photon spot on the sample. The two-stage zoom optics between sample and entrance aperture [‘1’ and ‘2’ in Fig. 4 ▸(*b*)] allow varying the (total) lateral magnification *M* in a wide range between *M* = 1 and 40. This wide range allows setting the filling angle of the HSA to any desired value between very small and large filling up to α_max_ = ±7°. In *k*-imaging mode the α^2^ term and the non-isochromaticity can be corrected numerically; see detailed discussion by Schönhense *et al.* (2020*b* ▸).

The behavior of *M* is governed by the Helmholtz–Lagrange invariant *M*sinα 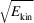 = const. Assuming that the full solid-angle range of 2π above the sample surface is ‘squeezed’ into a range of α_max_ = ±2.5° inside the HSA, we obtain magnification factors between *M* = 14 (for *E*_pass_ = 4 eV) and *M* = 1.4 (for *E*_pass_ = 400 eV). These values reflect the effects of the acceleration from the initial energy (here *E*_kin_ = 1.5 eV) to the pass energy and the ‘angular compression’ from 90° to 2.5°. In the general case the magnified image is cropped by the entrance aperture and hence the size of the photon beam at the sample plays an important role for the total transmission. For the interplay of *k*_||,max_, *E*_pass_, α_max_ and corresponding values of the non-isochromaticity, see equation (6) and Fig. 4 ▸ of Schönhense *et al.* (2020*b* ▸).

In the small-spot limit (for the present settings at beam diameters <17 µm) the magnified image lies completely inside the entrance aperture for both pass energies. Hence, there is no beam restriction by the entrance aperture and the total gain factor is just the number of resolved time slices, here *N* ≃ 50. In the limit of large beam diameters (>200 µm), the transmitted intensity is proportional to *E*_pass_. Then a second gain factor *N* is caused by the entrance aperture. In turn, the total gain factor is *N* in the small-spot limit and up to *N* ^2^ in the large-spot limit. Given a diameter of the laser beam of 50 µm, the present situation is an intermediate case. Due to the off-normal impact angle of 67.5° the laser beam yields a photon footprint on the sample surface of ∼130 µm × 50 µm. The magnified image of this photon footprint in the analyzer entrance plane is 180 µm × 70 µm for *E*_pass_ = 400 eV and 1800 µm × 700 µm for *E*_pass_ = 4 eV. For *E*_pass_ > 60 eV the image fits completely into the 0.5 mm entrance aperture, whereas for *E*_pass_ = 4 eV (*M* = 14) only ∼16% of the photoelectrons pass through the entrance aperture. For these conditions the total gain factor lies between 200 and 300 for *E*_pass_ between 8 and 4 eV, respectively. We notice that these restrictions do not exist for a pure ToF instrument, because there are no beam-confining apertures except the field aperture defining the ROI (see Section 2.6).

For an experimental verification, we studied the HSA + ToF hybrid mode at *E*_pass_ = 200 and 400 eV and slit *W* = 1 mm (α_max_ < 1°). The transmitted bandwidth is 440 meV at *E*_pass_ = 200 eV and 880 meV at 400 eV, which is sufficient to record the complete occupied part of the Tamm state of Re(0001) and of the Shockley state of Au(111) in one acquisition without scanning. Figs. 5 ▸(*g*)–5(*j*) show results in terms of *E*_B_-*versus*-*k*_*x*,*y*_ sections through the as-measured *I*(*k*_*x*_, *k*_*y*_, τ) data arrays (τ, time-of-flight), without correction of the linear transit time and parabolic α^2^ term showing up in the *k*_*y*_ sections (*h*, *j*). The dashed horizontal line marks the position of the Fermi edge. For 1T-TiTe_2_, recorded at *E*_pass_ = 400 eV (*k*, *l*), a bandwidth of 880 meV is acquired simultaneously. The count rate in this measurement with activated ToF booster was ∼3 × 10^6^ counts s^−1^; the data array was acquired in 10 min. Here we allowed a larger angular filling of α_max_ ≃ 2°, leading to a larger transit-time spread and α^2^ curvature, visible in (*k*) in the uncorrected section (top) and corrected by data processing (bottom). An additional curvature results from the very short ToF section (300 mm) used for these first experiments. The drift energy was set to *E*_drift_ = 9 eV, where we measured a temporal dispersion of 22 ps per 1 meV. The DLD thus resolves time slices of ∼18 meV width each. For comparison, the same data stack was recorded in the scanning mode at *E*_pass_ = 16 eV using 400 µm slit size, which also yields a resolution of ∼18 meV. In this mode the intensity was 20 kcounts s^−1^, *i.e.* the gain in efficiency was ∼150, in fair agreement with the theoretical expectation.

The energy resolution of the HSA without ToF booster has been determined at the Fermi edge of an Au film at various analyzer settings at sample temperatures of 30 and 15 K. The measured half widths (16–84%) of the Fermi-edge cutoff are given in Figs. 5 ▸(*m*)–5(*o*); for settings see figure caption. Deconvolution with the thermal broadening (3*kT* ≃ 10 and 5 meV for 30 and 15 K) yields the analyzer resolution Δ*E*_exp_, which can be compared with the expected values Δ*E*_theo_. For *E*_pass_ = 12 eV (slits *W* = 0.5 mm), Δ*E*_exp_ = 14.4 meV is close to Δ*E*_theo_ = 13.3 meV (*m*). For *E*_pass_ = 8 eV (*W* = 0.4 mm) the measured widths for the two temperatures [blue, 30 K; black 15 K in Fig. 5 ▸(*n*)] yield Δ*E*_exp_ = 7.8 and 7.5 meV, in good agreement with Δ*E*_theo_ = 7.1 meV. For the smallest slit (*W* = 0.2 mm) the value of Δ*E*_exp_ = 4.2 meV is still close to the expected value of Δ*E*_theo_ = 3.6 meV. The intensity profile Fig. 5 ▸(*o*) reveals a significant artifact slightly above *E*_F_ (marked by the yellow arrow). The inset shows that this intensity is not just a high-energy wing but can be located as a spurious additional intensity peak, separated from the Fermi edge. Upon further lowering of *E*_pass_ this spurious intensity increases and masks the true Fermi cutoff. Close inspection of the Gauss image reveals that this intensity is visible as a fringe around the image of the entrance aperture in the exit plane. This fringe might originate from a fine structure in the photon footprint due to reflections in the UV optics or from improper setting of the Jost-plate potentials. Clarifying this point would require ray-tracing calculations using a 3D model of the HSA including the details of the fringe field correction.

These first measurements validate that *k*-microscopy at high resolution is possible for sufficiently high photon flux in a small spot. The present resolution limit close to 4 meV FWHM has been determined for *E*_pass_ = 8 eV. The instrument allows *k*-imaging down to *E*_pass_ = 4 eV. Improvement of resolution by another factor of two seems possible, once the origin of the spurious intensity in the high-energy wing has been identified and eliminated. In the hybrid mode the energy-resolution limit is defined by the time resolution (180 ps for the present DLD). ToF-based *k*-imaging works well down to *E*_drift_ = 6 eV providing an energy resolution of 9 meV. The combination of the new generation of DLDs (≤100 ps resolution) with a longer drift tube should yield a resolution limit of Δ*E*_ToF_ < 5 meV. The spurious intensity is absent for the high pass energies used in the hybrid mode.

### 2.6. Sub-µm regions-of-interest selected by a field aperture

The high intensity of the UV laser [APE’s Levante Emerald ps OPO and HarmoniXX FHG (3+1) harmonic generator; https://www.ape-berlin.de/en/laser-arpes/] allowed studying the spatial-resolution limit in *k*-imaging mode, which is important for experiments with inhomogeneous or microstructured samples. Although a MM is optimized for high *k*-resolution it has also good imaging properties in PEEM mode. Here we will show that a ToF-MM facilitates *sub-micrometre-ARPES* without the need for a small photon spot. A field aperture in the first intermediate real-space image plane (Gaussian plane) selects the desired size and position of the analyzed region (ROI) on the sample surface, as sketched in Figs. 4 ▸(*a*) and 4(*b*). There is one issue here: high lateral resolution in PEEM mode (typically in the 10–50 nm range) demands a small contrast aperture, confining the angular (more precisely *k*_||_) acceptance. However, this confinement is in conflict with momentum imaging, where a certain *k*-range must be transmitted by the microscope. In turn, high spatial resolution (*i.e.* a small ROI) competes with the need to image the *k*-range of interest.

The limiting factor is the spherical aberration of the objective lens, which leads to a deterioration of the lateral resolution by δ_s_ = *c*_s_α^3^ (Hawkes, 2009 ▸). Here α is the entrance angle of the electrons into the lens system after acceleration by the extractor. For MMs this expression is translated into momentum coordinates, yielding δ_s_ = *c*_s_^*k*^ *k*_||_^3^ (Tusche *et al.*, 2015 ▸). We can estimate the expected resolution by considering just the homogeneous accelerating field of the extractor. Bauer (1985 ▸) and later Tromp (2011 ▸) derived equations for the aberration coefficient of a uniform field *F* at the sample surface, showing that *c*_s_ ∝ *E*_kin_/*eF*. Given the maximum kinetic energy *E*_kin_^max^ = 2.5 eV in the UV-laser experiment, these equations suggest resolution limits of δ_s_ ≃ 500 and 330 nm for fields of *F* = 5 and 7.5 kV mm^−1^, respectively. However, this simple estimation neglects the contributions of the spherical aberrations of the lenses. It is thus not clear whether the ‘Nano-ARPES’ regime is really accessible just by using a sufficiently small field aperture, regardless of the size of the photon footprint on the sample. The aberration of the aperture lens (the hole in the extractor with different field strengths at both sides) is negligible (Wang *et al.*, 1993 ▸) but the subsequent lenses have non-vanishing aberrations that contribute to the total resolution. We are not aware of previous attempts to validate this prediction experimentally.

**Figure 6:**
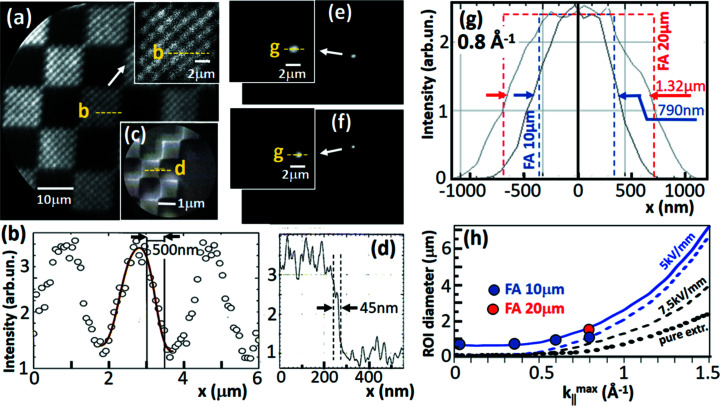
Small-area momentum microscopy using small field apertures. Results taken for an Au checkerboard structure (‘Chessy’, Plano GmbH) with fully open field aperture (*a*) and apertures of 20 µm (*e*) and 10 µm (*f*). (*c*) Resolution limit in PEEM mode. (*b*, *d*, *g*) Line scans along the dashed lines in (*a*, *c*, *e* and *f*). Dashed square profiles in (*g*) denote the widths expected for an ideal, aberration-free lens. (*a*, *b*, *e*–*g*) Measured at the Fermi edge of the Au structure at *E*_kin_ = 2.5 eV, corresponding to 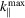 = 0.8 Å^−1^. (*h*) ROI diameter as a function of 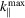 as measured for field apertures of 20 µm (red dot) and 10 µm (blue dots) and as calculated for the full lens optics at field *F* = 5 kV mm^−1^ in the small-aperture limit (dashed blue curve) and for a 10 µm aperture (full blue curve). For comparison, the small-aperture limits for *F* = 7.5 kV mm^−1^ and for the pure extractor field of 5 kV mm^−1^ are also shown [all curves from Tusche *et al.* (2015 ▸)].

This interplay of accepted *k*-field radius 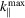 and achievable spatial resolution (*i.e.* the actual size of the ROI) has been elucidated exploiting the high photon flux of the UV-laser. An Au on Si microstructure (‘Chessy’, Plano GmbH) consisting of 1 µm × 1 µm squares grouped in a hierarchy of checkerboard patterns with 10 µm × 10 µm arrays served as a test sample. Fig. 6 ▸ shows Gaussian images with open contrast aperture as needed for the *k*-imaging mode and open field aperture (*a*), and with a small contrast aperture as needed for the PEEM mode (*c*). Line scans along the dashed lines in (*a*, *c*) reveal spatial resolutions of ∼500 nm in the *k*-imaging mode (*b*) and ∼45 nm for the PEEM mode (*d*). The present microscope has an array of nine selectable and adjustable field apertures with sizes down to 10 µm diameter. For identical settings of the microscope like in the full-field image (*a*), we measured the Gaussian images of the 20 µm and 10 µm apertures. The details of 10 µm × 10 µm shown in Figs. 6 ▸(*e*) and 6(*f*) indicate that the areas accepted by the apertures have sizes in the order of one 1 µm × 1 µm square. Fig. 6 ▸(*g*) shows horizontal line scans through the detail images in (*e*, *f*), revealing full widths at half-maximums (FWHM) of 1.3 and 0.8 µm for the 20 and 10 µm field apertures, respectively.

This study was performed at 20 kV extractor potential (4 mm gap) and a magnification factor of *M* = 14 between sample and first Gaussian image. Assuming an ideal lens (without aberrations), the apertures of 20 and 10 µm translate into ROI diameters on the sample of 1.4 and 0.7 µm, indicated as dashed square profiles in (*g*). The geometric sizes show good agreement with the measured FWHM values. In particular, there is a clear decrease of the effective size of the ROI when changing from the 20 µm to the 10 µm aperture, with the latter still showing a flat top. The Gaussian broadening of the rims of the measured profiles is a result of the spherical aberration.

Measurements (*a*, *b*, *e*, *f* and *g*) have been taken at the maximum possible kinetic energy of 2.5 eV, corresponding to a photoemission horizon (defined by the emission angle θ = 90°) of 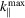 = 0.8 Å^−1^. In order to study how the ROI size varies with 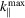 , line profiles like Figs. 6 ▸(*e*)–6(*g*) have been taken at *E*_kin_ = 2.5, 1.5, 0.5 and ∼0 eV, corresponding to 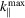 = 0.8, 0.6, 0.35 and ∼0 Å^−1^, respectively. These line scans (not shown) reveal FWHM values of 790, 770, 740 and 730 nm, *i.e.* the effective ROI size decreases slightly with decreasing 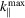 as expected for the behavior of the spherical aberration. In Fig. 6 ▸(*h*) the measured widths (dots) are compared with simulated results of Tusche *et al.* for the present lens geometry (Tusche *et al.*, 2015 ▸). The dashed blue curve shows the simulation for 5 kV mm^−1^ extractor field in the small-aperture limit. For comparison with the experimental results we have added the geometric projection of the 10 µm aperture for *M* = 14, yielding the full blue curve in Fig. 6 ▸(*h*). The measured points compare fairly well with this curve, *i.e.* with the sum of geometric size and aberration term. The limited horizon at this small photon energy does not allow to track the increase of the aberration term with 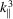 , which is an unavoidable property of all electron lenses [it is worth reading Hawkes (2009 ▸) in this context]. Theoretically, the aberration drops with increasing extractor field: for 7.5 kV mm^−1^ the δ_s_ term is already reduced by a factor of two and for the pure extractor field of 5 kV mm^−1^ without objective lens by a factor of three, shown as dashed and dotted black curves, respectively, in Fig. 6 ▸(*h*).

The results of Fig. 6 ▸ give rise to several important conclusions. *Sub-micrometre momentum microscopy* is feasible with the present (standard) low-energy electron optics, regardless of the size of the photon footprint. A sufficiently small field aperture and high photon intensity are mandatory. The space-charge effect may set a practical limit if the illumination spot is too large in comparison with the ROI (Schönhense *et al.*, 2021 ▸). The measured spot profiles suggest a further decrease of the selected area using a 5 µm aperture, which would yield a ROI size of <500 nm. The limiting factor is the *k*-field diameter and not the kinetic energy. Up to 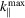 ≃ 1 Å^−1^ the ROI can be ≤1 µm. At larger *k*-acceptance the spherical aberration (increasing with 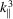 ) shows a steep rise, which sets a lower limit to the size of the ROI, regardless of the aperture. For 7.5 kV mm^−1^ extractor field and *k*-field radii of 1.5 and 2 Å^−1^ the aberration disk has diameters of 4 and 10 µm, respectively.

Experiments with the same electron optics at higher energies shine further light into this possibility of small ROI diameters. The Okinawa group works with HHG radiation (*h*ν = 22 eV) (Madéo *et al.*, 2020 ▸; Man *et al.*, 2021 ▸) and observed a clipping of the rim of the *k*-horizon when using small field apertures. With 10–12 kV extractor and 10 µm aperture the visible *k*-horizon had a radius of ∼1 Å^−1^, in accordance with Fig. 6 ▸(*h*). Moreover, the transmission of the apertures drops more rapidly than expected from their areas. Both observations are signatures of the spherical aberration. With increasing emission angle from the sample, the rays are overfocused and cross the optical axis with increasing distance from the focal plane. The small aperture in the focal plane blocks these large-angle rays, showing up as clipping of the *k*-horizon and strong dropping of transmitted intensity. The Stony Brook group works with a cavity-enhanced HHG source (similar photon energy) running at 60 MHz, thus keeping space-charge effects negligible (Corder *et al.*, 2018 ▸). Working at 7 kV extractor with small field apertures they observed attenuation of the outer *k*_||_ regions. Pump–probe experiments on graphite with 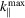 ≃ 2 Å^−1^ and 10 µm ROI were successful for ≤10 µJ cm^−2^ excitation fluence and 40 µm × 60 µm photon footprint; several papers are in progress (*e.g.* Kunin *et al.*, 2021 ▸). Graphite is particularly challenging due to the short excited-state lifetime and large BZ.

For such cases with large 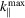 there is the interesting possibility of displacing the *k*-confining aperture (contrast aperture in PEEM mode) off-center. This enables the selection of a certain off-normal *k* region, *e.g.* in the vicinity of a *K* and *K*′ point. Simulations reveal that off-normal *k*-fields with diameters up to ∼2 Å^−1^ can be recorded with ∼1 µm ROI size, smaller *k*-fields with sub-µm ROI size, provided the interplay of field aperture and *k* aperture is optimized. With increasing distance from the center (*k*_||_ = 0), the excitation of the objective lens must be reduced, such that the image of the desired region, *e.g.* the *K*-points, stays in best focus. We are not aware of experiments exploiting this possibility. *Sub-micrometre ARPES* utilizing the real-space imaging capabilities of MMs is still largely unexplored.

We conclude this section by considering possible influences of the space-charge interaction in the pulse-picker and bandpass modes of operation. The Coulomb repulsion between the electrons can cause energy shifts and signal broadening. For photoemission experiments at intense pulsed sources like FELs and HHG sources this effect poses the most serious limitation of overall performance. In classical PEEMs and MMs the high extractor field pulls all electrons (in particular the large amount of slow secondary electrons) into the lens optics, where they can interact over flight paths of tens of mm. Space-charge effects in such instruments have been studied in detail at various photon sources [BESSY (Schönhense *et al.*, 2015*b* ▸), PETRA-III (Schönhense *et al.*, 2018 ▸) and at the free-electron laser FLASH (Schönhense *et al.*, 2021 ▸)]. In these papers schemes for *a posteriori* data correction or *a priori* suppression are proposed and used. The present paper deals with photon sources with high pulse rates, where only a few photoelectrons per pulse are emitted (even in the pulse-picking mode at BESSY, Section 2.3). In turn, the space-charge effect was insignificant in the measurements shown. Only in the laser experiment with small ROI (Fig. 6 ▸) we observed the onset of space charge shifts, because just a small fraction of the total photocurrent is recorded. For a ROI set to 1 µm diameter and the given photon footprint of 40 µm × 100 µm, the total number of emitted electrons exceeds the recorded fraction by more than three orders of magnitude. Since all emitted electrons contribute to the space-charge effect, the onset of a shift appeared already in the range 10^5^ counts s^−1^ (total emitted electrons: 4 × 10^8^ s^−1^). At low photon energies the retarding mode of the front lens (Schönhense *et al.*, 2021 ▸) cannot be used. However, the situation would be strongly improved by a smaller photon spot, thus reducing the fraction of undesired electrons from outside of the ROI. Comparing different strategies with respect to space charge, using band-pass preselection one can potentially increase the photon intensity (if possible) until the space charge limitation sets in, while the maximum detector count rate is not a limiting factor. In contrast, the chopping mode, besides requiring radiation-resistive samples, poses a limit of the maximum detector count rate.

### 2.7. Displaced ‘island-orbit’ filling pattern with 5 MHz

Different from the previous methods, which do not affect the storage ring, there is another possibility of implementing ToF into the multi-bunch operation scheme that was tested at BESSY II. It is based on a special storage ring setting, called TRIBs (transverse resonance island buckets), generating a second stable orbit, wiggling around the main orbit in the equatorial plane (Goslawski *et al.*, 2019 ▸). The island orbit can be populated with a single bunch or a few-bunch filling pattern offering reduced repetition rates compared with the multi-bunch filling on the main orbit. Since the island orbit differs in transverse position and angle from the main orbit, it provides an additional well separated radiation source in bending magnets and undulators, which in turn can be selected via a pinhole shifted off the equatorial plane, blocking the radiation from the multi-bunch train. The subsequent monochromator optics corrects for this shift, so that the spot in the exit plane stays the same. Currently, at BESSY II the Top-Up injection in this ‘Two-Orbit/TRIBs’ mode is under optimization and the bunch current stored on the island orbit is increased, reaching values compared with the Camshaft bunch of the standard hybrid filling (3–4 mA). Beyond this application, as bunch separation scheme, offering two different repetition rates simultaneously, the TRIBs setting allows for generating synchrotron radiation with properties not accessible so far. Within a proof-of-principle experiment it was shown that TRIBs enables MHZ-fast helicity flipping of X-rays from an undulator, more than three orders of magnitude faster than state-of-the-art technologies (Holldack *et al.*, 2020 ▸). In particular, X-ray circular dichroism (XMCD), one of the main spectroscopic tools to study magnetism, will benefit enormously from this development. The TRIBs/Two-Orbit mode is now offered regularly in Top-Up user operation for one week per year at BESSY II.

Due to the high solid-angle acceptance of the U125-2 NIM beamline, it is possible to image the horizontally offset source point of the ‘island-orbit’ in the undulator on the intermediate focus after the first mirror, also horizontally separated from the main beam. By detecting the different pulse rates of the orbits at the experiment, it was possible to insert a horizontal aperture in front of the main beam until only photons with a pulse rate corresponding to that of the island orbit of 5 MHz are allowed to pass through the further beamline up to the experiment. This did not require any further optimization of the alignment of the optical elements of the beamline. It shows that it is possible to use the two sources separately at the U125-2 NIM, even in simultaneous operation of the orbits (‘twin orbit mode’).

**Figure 7:**
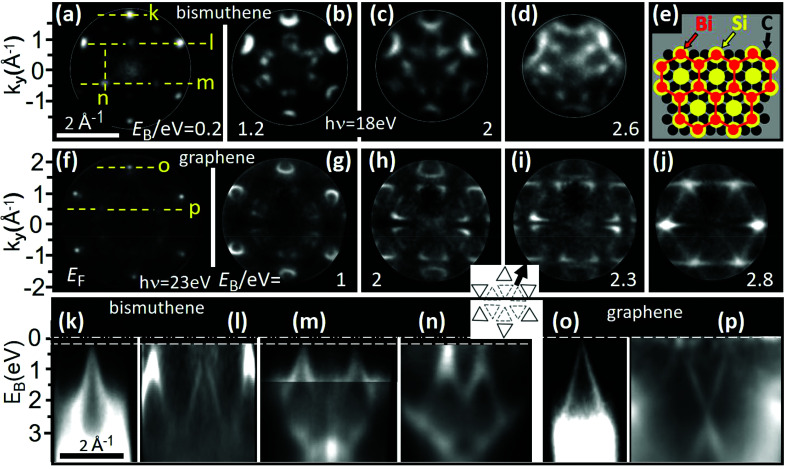
Momentum patterns of bis­muthene and zero-layer graphene on SiC(0001), recorded at the U125-2_NIM of BESSY II. Results have been recorded for bis­muthene in a special ‘island-orbit’ filling mode of the storage ring, in which four bunches travel on an orbit oscillating around the equatorial plane and for graphene in few-bunch mode. (*a*–*d*) *k*_*x*_–*k*_*y*_ sections through a data array *I*(*E*_B_,*k*_*x*_,*k*_*y*_), recorded at *h*ν = 18 eV. The valence-band maximum lies 0.2 eV below *E*_F_ (*a*); the opening of the cones with increasing binding energy is visible in (*b*–*d*). (*e*) Structure of bis­muthene on SiC(0001). (*f*–*g*) Analogous results for zero-layer graphene, recorded at *h*ν = 23 eV with few-bunch filling of BESSY II. The Dirac point lies at *E*_F_ (*f*) and the opening cones show the well known triangular cross sections (*g*–*j*). For this sample a (2 × 2) superstructure is present, sketched as dashed triangles in the inset in (*i*). (*k*–*p*) *E*_B_-*versus*-*k*_*x*_ sections showing the linear band dispersions for bis­muthene (*k*–*n*) and graphene (*o*, *p*). Panels (*m*, *n*) show the cones of the first BZ and (*p*) shows the ‘mirrored cones’ of the (2 × 2) structure.

The ToF momentum microscope has been used in a test beam time at the U125-2_NIM using a four-bunch filling pattern on an island orbit coexisting with the common multi-bunch pattern on the main orbit. A result measured in this mode is shown in Fig. 7 ▸. The sample was a bis­muthene monolayer on SiC(0001). This novel material was recently synthesized by epitaxial growth on a hydrogen-etched SiC wafer and studied by STM/STS and photoemission (Reis *et al.*, 2017 ▸). A band structure similar to graphene but with a large spin–orbit induced total energy gap of ∼0.8 eV makes bis­muthene on SiC a promising candidate for a room-temperature quantum spin Hall system.

Figs. 7 ▸(*a*)–7(*d*) show *k*_*x*_–*k*_*y*_ momentum sections for bis­muthene at the valence-band maximum 0.2 eV below *E*_F_ and at larger *E*_B_ as given in the panels. Close inspection of (*a*) reveals an inner ring of six weak spots (corresponding to the first BZ) and an outer ring of six bright spots (repeated BZ). With increasing *E*_B_ the spots open up to threefold-deformed circles with pronounced intensity variations along their periphery. The broadening of the circular patterns at the *K* points is caused by the spin–orbit induced band splitting (Reis *et al.*, 2017 ▸). The sketch in Fig. 7 ▸(*e*) shows the bis­muthene structure, a honeycomb lattice in perfect registry with the SiC(0001) substrate. The centers of the Bi-hexagons (red) form a 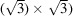 R30° superstructure in the on-top position of the topmost Si layer.

The second row in Fig. 7 ▸ [panels (*f*)–(*j*)] shows the corresponding *k*_*x*_–*k*_*y*_ patterns for zero-layer graphene on SiC(0001), recorded in a few-bunch filling pattern of BESSY II on the main orbit as described by Tusche *et al.* (2016 ▸). The Dirac point lies at the Fermi energy (*f*), and with increasing *E*_B_ the Dirac cones open and show their characteristic triangular shape (*g*–*j*). This sample showed a (2 × 2) superstructure, sketched in the inset in (*i*). In addition to the well known six Dirac cones (full triangles), a second ring of Dirac cones with mirrored cross section (dashed triangles) appears. In the sequence (*g*,*h*,*i*) the triangles grow and finally touch each other in (*j*). Note the striking similarity of the patterns for bis­muthene and zero-layer graphene at 1 eV below the valence band maximum/Dirac point, (*b*) and (*g*), respectively.

The bottom row (*k*–*p*) shows *E*_B_-*versus*-*k*_*x*,*y*_ sections along the dashed lines marked in (*a*) and (*f*). For bis­muthene, (*k*) shows a cut through a single cone, (*l*) through two cones of the outer ring (second BZ) and one on the inner ring (first BZ). Panels (*m*) and (*n*) show cuts through two cones of the first BZ in perpendicular orientations along *k*_*x*_ (*m*) and *k*_*y*_ (*n*). For zero-layer graphene, panel (*o*) shows a cut through one of the Dirac cones of the bright outer ring and (*p*) through two cones of the inner (mirrored) ring of the (2 × 2) superstructure as marked in (*f*).

The bis­muthene example proves the usability of the island-orbit mode (coexisting with normal multi-bunch filling on the main orbit) for ToF experiments. The better *k*-resolution in the graphene data reflects the higher geometric quality of this film and is not related to the different acquisition modes. For the physics of bis­muthene and details on the graphene measurement, see Reis *et al.* (2017 ▸) and Tusche *et al.* (2016 ▸).

## 3. Conclusions and outlook

Three-dimensional photoelectron recording schemes in angular- or momentum-resolving ToF instruments exploiting the time structure of synchrotron radiation or pulsed lasers have proven superior in many respects. Parallel acquisition of many energies minimizes the radiation dose for sensitive samples (Giangrisostomi *et al.*, 2018 ▸), reduces the data-acquisition time in photon-hungry experiments in the soft- (Medjanik *et al.*, 2017 ▸) and hard-X-ray range (Medjanik *et al.*, 2019 ▸), and enables full-field XPD (Fedchenko *et al.*, 2019 ▸). First studies indicate that the latter method has a huge potential for *in situ* structural analysis (Fedchenko *et al.*, 2020 ▸; Medjanik *et al.*, 2021 ▸; Hoesch *et al.*, 2021 ▸), in particular in the ultrafast regime at FELs (Kutnyakhov *et al.*, 2020 ▸; Dendzik *et al.*, 2020 ▸; Curcio *et al.*, 2021 ▸; Pressacco *et al.*, 2021 ▸). XPD measurements are an indispensable prerequisite for aberration-free bulk band mapping using hard X-rays (Elmers *et al.*, 2020*a* ▸; Medjanik *et al.*, 2021 ▸; Babenkov *et al.*, 2019 ▸; Schönhense *et al.*, 2020*a* ▸). In combination with an imaging spin filter, the 3D-recording architecture enables accessing a full spin texture in reasonable time (Elmers *et al.*, 2016 ▸, 2020*b* ▸; Schönhense *et al.*, 2017 ▸; Vasilyev *et al.*, 2020 ▸; Chernov *et al.*, 2021 ▸). All these experiments used special filling patterns of storage rings [or a mechanical chopper in Giangrisostomi *et al.* (2018 ▸)], because the short time gap in normal multi-bunch operation (pulse rates between 100 and 500 MHz) is prohibitive for ToF energy discrimination. ToF-based angular- or momentum-resolved photoelectron spectroscopy is superior to 2D recording techniques but suffers from the rare availability of special few-bunch filling patterns of storage rings.

This paper describes two loopholes out of this dilemma. The interplay of desired energy resolution for a certain bandwidth and time resolution of the detector dictates the length of the required time interval. Given present detector resolutions of ≤100 ps, time intervals of >100 ns allow resolving about 1000 time slices in the selected energy band of several eV width, which is sufficient for most applications. The first approach is based on a high-frequency deflector unit between two pinholes in the ray path of a momentum microscope, acting as efficient pulse picker. The deflector is phase-locked to the bunchmarker of the storage ring such that it can select a periodic train of electron pulses at a lower repetition rate and correspondingly larger time gap for the ToF analysis. We show examples of ‘picking’ a convenient rate of 5 MHz electron pulses excited by the 100 MHz photon-pulse train of MAX II. Similarly, the camshaft pulse (with enhanced bunch charge) at 1.25 MHz rate was picked from the 500 MHz multi-bunch train in the hybrid mode of BESSY II. The gain due to the ToF recording scheme is higher than the loss induced by the duty cycle of the blanking unit. A dedicated instrument capable of selecting any desired pulse rate from the filling pattern (200 MHz) is being developed for PETRA III/PETRA IV. The chopping mode uses only a fraction of the photon pulses, given by the duty cycle (*e.g.* 1/20 for the example shown in Section 2.2). In turn, the radiation load at the sample is higher by the inverse factor, which may hamper the study of sensitive samples. Similarly, charging of low-conductance samples may cause undesired energy shifts. This duty-cycle factor gets worse with increasing photon pulse rate. In spite of these disadvantages with sensitive or charging samples, the benefit of the pulse-picking mode is that it enables ToF-MM experiments without special demands for the storage-ring operation.

The second approach retains the full pulse rate but reduces the bandwidth of the electron spectrum being dispersed in the ToF low-energy drift section by a bandpass pre-filter. In this mode there is no enhanced radiation load or surface charging. Bandwidth reduction in the 10 eV range can be achieved by a high-pass filter lens in the microscope column as described by Tusche *et al.* (2016 ▸). However, this is not sufficient for pulse rates of ≥100 MHz. Moreover, high-pass filtering does not allow to select a certain core level because the faster electrons from the valence range or higher-lying core levels would reach the detector as well. For general applications an energy-dispersive element needs to be implemented in the microscope column. We discussed in detail the possibilities opened up with a hemispherical analyzer combined with ToF recording of the transmitted energy band. Fourier-plane imaging in a momentum microscope overcomes the strong variation of the transit time for different Kepler ellipses in the analyzer (Imhof *et al.*, 1976 ▸; Sise & Zouros, 2015 ▸). Using the new generation of delay-line detectors (≤100 ps resolution), the ToF section can resolve between *N* = 20 and 100 time slices for pulse rates between 500 and 100 MHz, respectively. For this mode we can define an effective ‘figure-of-merit’ as the gain in recording efficiency when switching from the classical 2D mode of an HSA to the 3D recording scheme of the *HSA-plus-ToF* hybrid mode. This gain is *N* in the limit of small photon footprints and increases to *N* ^2^ in the limit of large photon footprints. In the latter case the transmission of the analyzer entrance lens increases by *N*, in addition to the parallel recording of *N* slices (Schönhense *et al.*, 2020*b* ▸). In comparison with hemisphere-based momentum microscopes (Tusche *et al.*, 2015 ▸, 2019 ▸) the gain factor is just given by the number of resolved time slices *N*. A prototype instrument with a large hemispherical analyzer (225 mm path radius) will be installed at Diamond [beamline I09 (Lee & Duncan, 2018 ▸)].

Although they have the same goal, the two methods were developed under different aspects. Pulse-picking in the time domain via electron-optical blanking is an *enabling technique*, opening up a previously closed avenue to efficient 3D photoemission recording in standard operation modes of storage rings. The hemisphere-plus-ToF hybrid instrument was developed under the aspect of *resolution and transmission improvement* by implementing a ‘ToF-booster’ behind a single-hemisphere momentum microscope. This microscope could be operated without the ToF, although with lower recording efficiency.

The present measurements validate that *k*-microscopy at high spatial and energy resolution is possible for sufficient photon flux. Using a narrow-band UV laser (80 MHz, <0.5 meV bandwidth) a resolution of Δ*E* = 4.2 meV FWHM has been measured for *E*_pass_ = 8 eV (presently limited by an artifact signal). The present setup allows *k*-imaging down to *E*_pass_ = 4 eV. Hence a further improvement of resolution by a factor of two seems possible, after some work on imaging quality and illumination spot. In the hybrid mode at high pass energies the energy-resolution limit is defined by the temporal dispersion in the drift section and the time resolution of the detector. Momentum imaging works well down to *E*_drift_ = 6 eV in the ToF section, resulting in an energy resolution of 9 meV. For an optimized DLD and longer drift tube we expect a resolution of Δ*E*_ToF_ < 5 meV.

The high intensity of the UV laser allowed elucidating the *spatial resolution in *k*-imaging mode* by means of field apertures. Using a calibrated test object, we measured a ROI size on the sample surface of 790 nm for a *k*-field of 1.6 Å^−1^ diameter. Results for different field apertures and different *k*-fields suggest that this ROI size is not yet aberration-limited. For the given (low-energy) microscope optics, ROIs in the 500 nm range are realistic for sufficiently small *k*-fields, enabling ‘sub-µm ARPES’ just by electron-optical confinement using a small field aperture. As long as space-charge effects (Schönhense *et al.*, 2015*b* ▸, 2018 ▸, 2021 ▸) are not the limiting factor, the photon spot size is not crucial; however, the photon density in the ROI must be sufficient for measurements in reasonable time. Given the conditions at high-brilliance beamlines like P04 (Viefhaus *et al.*, 2013 ▸) or P22 (Schlueter *et al.*, 2019 ▸) at PETRA III (photon spots of 10–20 µm with Gaussian profile), a reduction of the ROI to 1 µm (placed in the maximum of the Gaussian) would cost ≥2 orders of magnitude in intensity. The resulting count rates would still allow MM at reduced data throughput. The Heisenberg uncertainty relation, Δ*k*Δ*x* < 1, ultimately limits the product of momentum and spatial resolution. The experimentally achieved value of Δ*k*Δ*x* ≃ 50 (0.01 Å^−1^ × 500 nm) still bears room for improvement. The limiting factor in a full-field imaging setup is the spherical aberration of the objective lens. The main advantage of this approach is its ease of use by switching from standard operation with full photon spot to small-area MM by just changing the field aperture. In order to enable optimized settings of the resolution for certain *k*-field diameters, the microscope must be equipped with an appropriate set of *k*-confining apertures. The *k*-aperture can be placed off-center, in order to reach large values of *k*_||_.

We conclude with a technical and a scientific outlook. Since it does not downgrade radiation sensitivity or surface charging, the bandwidth pre-selection mode appears more versatile. However, the combination of a *k*-imaging hemisphere with ToF recording is rather complex. Bandwidth pre-selection in the energy domain can be achieved by simpler means. A resolution of 30 meV (typical photon bandwidth of a soft-X-ray beamline) at a 100 MHz storage ring and a DLD with 80 ps resolution (125 resolved time slices) would require narrowing the energy bandwidth to 3.8 eV. This can be done by a much simpler dispersive bandpass filter on the basis of a multipole-deflector (asymmetric dodecapole) doublet in an almost straight microscope column. In this multipole filter, the deflector field is superimposed to the dodecapole field with its well known property of image-aberration correction up to third order (Matsuo *et al.*, 1982 ▸; Boerboom *et al.*, 1985 ▸), thus retaining a high-quality image. Such a filter has been successfully used at the hard X-ray beamline P22 of PETRA III.

As a scientific outlook, we address the technique of momentum mapping of electron pairs that are detected in coincidence. This 2e spectroscopy provides a direct insight into two-particle correlations; for an excellent overview, see the recent review by Schumann *et al.* (2020 ▸). Two classes of processes have been studied – double photoelectron emission (*h*ν, 2e) and pair emission after fast electron impact (e, 2e). A spin-resolved variant gives access to the exchange interaction and spin entanglement in such processes (Vasilyev *et al.*, 2017 ▸). Since the delayline detector can record multiple events (see https://www.surface-concept.com/downloads/info/ml_dld.pdf), coincidence spectroscopy will benefit substantially from the parallel imaging technique. We may speculate that this advantage also applies to other types of coincidence experiments, like the (e, e + *h*ν) experiment performed by Jannis *et al.* (2021 ▸).

*Note added in proof.* The small-ROI mode has recently been exploited for resolving sub-micrometre-sized single antiferromagnetic domains observing antiferromagnetic parity violation in the electronic structure of Mn_2_Au (Fedchenko *et al.*, 2021 ▸).
